# Impact of nine common type 2 diabetes risk polymorphisms in Asian Indian Sikhs: *PPARG2 (Pro12Ala)*, *IGF2BP2*, *TCF7L2 *and* FTO *variants confer a significant risk

**DOI:** 10.1186/1471-2350-9-59

**Published:** 2008-07-03

**Authors:** Dharambir K Sanghera, Lyda Ortega, Shizhong Han, Jairup Singh, Sarju K Ralhan, Gurpreet S Wander, Narinder K Mehra, John J Mulvihill, Robert E Ferrell, Swapan K Nath, Mohammed I Kamboh

**Affiliations:** 1 Department of Pediatrics, University of Oklahoma Health Sciences Center, Oklahoma City, OK, USA; 2 Oklahoma Medical Research Foundation, Oklahoma City, OK, USA; 3 Guru Nanak Dev University, Amritsar, Punjab, India; 4 Hero DMC Heart Institute, Ludhiana, Punjab, India; 5 All India Institute of Medical Sciences, New Delhi, India; 6 Department of Human Genetics, Graduate School of Public Health, University of Pittsburgh, Pittsburgh, PA, USA; 7 Department of Pediatrics, Section of Genetics University of Oklahoma Health Sciences Center, 975 NE 10th, BRC-254A, Oklahoma City, OK – 73104, USA

## Abstract

**Background:**

Recent genome-wide association (GWA) studies have identified several unsuspected genes associated with type 2 diabetes (T2D) with previously unknown functions. In this investigation, we have examined the role of 9 most significant SNPs reported in GWA studies: [peroxisome proliferator-activated receptor gamma 2 (*PPARG2*; rs 1801282); insulin-like growth factor two binding protein 2 (*IGF2BP2*; rs 4402960); cyclin-dependent kinase 5, a regulatory subunit-associated protein1-like 1 (*CDK5*; rs7754840); a zinc transporter and member of solute carrier family 30 (*SLC30A8*; rs13266634); a variant found near cyclin-dependent kinase inhibitor 2A (*CDKN2A*; rs10811661); hematopoietically expressed homeobox (*HHEX*; rs 1111875); transcription factor-7-like 2 (*TCF7L2*; rs 10885409); potassium inwardly rectifying channel subfamily J member 11(*KCNJ11*; rs 5219); and fat mass obesity-associated gene (*FTO*; rs 9939609)].

**Methods:**

We genotyped these SNPs in a case-control sample of 918 individuals consisting of 532 T2D cases and 386 normal glucose tolerant (NGT) subjects of an Asian Sikh community from North India. We tested the association between T2D and each SNP using unconditional logistic regression before and after adjusting for age, gender, and other covariates. We also examined the impact of these variants on body mass index (BMI), waist to hip ratio (WHR), fasting insulin, and glucose and lipid levels using multiple linear regression analysis.

**Results:**

Four of the nine SNPs revealed a significant association with T2D; *PPARG2 *(Pro12Ala) [odds ratio (OR) 0.12; 95% confidence interval (CI) (0.03–0.52); p = 0.005], *IGF2BP2 *[OR 1.37; 95% CI (1.04–1.82); p = 0.027], *TCF7L2 *[OR 1.64; 95% CI (1.20–2.24); p = 0.001] and *FTO *[OR 1.46; 95% CI (1.11–1.93); p = 0.007] after adjusting for age, sex and BMI. Multiple linear regression analysis revealed significant association of two of nine investigated loci with diabetes-related quantitative traits. The 'C' (risk) allele of *CDK5 *(rs 7754840) was significantly associated with decreased HDL-cholesterol levels in both NGT (p = 0.005) and combined (NGT and T2D) (0.005) groups. The less common 'C' (risk) allele of *TCF7L2 *(rs 10885409) was associated with increased LDL-cholesterol (p = 0.010) in NGT and total and LDL-cholesterol levels (p = 0.008; p = 0.003, respectively) in combined cohort.

**Conclusion:**

To our knowledge, this is first study reporting the role of some recently emerged loci with T2D in a high risk population of Asian Indian origin. Further investigations are warranted to understand the pathway-based functional implications of these important loci in T2D pathophysiology in different ethnicities.

## Background

Type 2 diabetes (T2D) is a common disease characterized by insulin resistance and reduced insulin secretion. It has become a health problem world-wide and the underlying molecular mechanisms involved in the development of diabetes remain poorly understood. Linkage and candidate gene studies have been highly successful in identifying genes for monogenic and syndromic forms of diabetes [1]. However, this approach has yielded limited results in mapping the genes for T2D [2-4]. The latest success of the genome-wide association (GWA) studies using high density SNPs across the genome and complex genetic analyses have identified several genes with previously unknown functions [5-9].

In this investigation, we have examined the role of nine most significant loci previously reported to be associated with T2D in Caucasian populations [5,6,10-12] in a diabetic case-control cohort of Khatri Sikhs obtained from North India. Our study is focused on the peroxisome proliferator-activated receptor gamma 2 (*PPARG2*; Pro12 Ala; rs1801282), insulin-like growth factor two binding protein 2 (*IGF2BP2*; rs 4402960), cyclin-dependent kinase 5, a regulatory subunit-associated protein 1-like 1 (*CDK5*; rs 7754840), a zinc transporter and member of solute carrier family 30 (*SLC30A8*; rs 13266634), a variant found near cyclin-dependent kinase inhibitor 2A (*CDKN2A*; rs 10811661), hematopoietically expressed homeobox (*HHEX*; rs 1111875), transcription factor-7-like 2 (*TCF7L2*; rs 10885409), potassium inwardly rectifying channel subfamily J member 11 (*KCNJ11*; rs 5219), and a recently discovered fat mass obesity-associated gene (*FTO*; rs 9939609). *TCF7L2 *gene is the most frequently replicated adult-onset T2D susceptibility gene [8]. Some of the *TCF7L2 *variants have been found to strongly associated with T2D in Asian Indian populations from South [13,14] and South West India [14], and also our North Indian cohort [15]. In this study, we have used the most significant *TCF7L2 *SNP associated with T2D in Sikhs for comparisons. Since the replication of positive association in multiple independently ascertained datasets from different ethnic groups is crucial for identifying the true population-specific susceptibility variants, we have examined the role of polymorphisms of these nine genes in our diabetes case-control cohort of Asian Indian Sikhs.

## Methods

### Human Subjects

The study subjects are part of the ongoing Sikh Diabetes Study (SDS) [16]. The focus of this study is on an endogamous community of Khatri Sikhs living in the Northern states of India, including Punjab, Haryana, Himachal Pradesh, Delhi, and Jammu & Kashmir. The DNA and serum samples of 532 T2D cases (299 male, 233 female) and 386 normal glucose tolerant (NGT) (184 male, 202 female) subjects were used in this investigation. Of the 532 cases, 324 are from family material (one index case from each family) and the remaining 208 are unrelated T2D cases from the same Sikh community. The cases were 25 or older with mean age at the time of recruitment (mean ± SD) of 54.2 ± 11.0 yrs. The diagnosis of T2D was confirmed by scrutinizing medical records for symptoms, use of medication, and measuring fasting glucose levels following the guidelines of American Diabetes Association [17]. A medical record indicating either (1) a fasting plasma glucose level ≥126 mg/dl or ≥7.0 mmol/l after a minimum12-h fast or (2) a 2-h post glucose level [2-h oral glucose tolerance test (OGTT)] ≥200 mg/dl or ≥11.1 mmol/l on more than one occasion with symptoms of diabetes. IGT was defined as a fasting plasma glucose level ≥100 mg/dl (5.6 mmol/l) but ≤126 mg/dl (7.0 mmol/l) or a 2-h OGTT ≥140 mg/dl (7.8 mmol/l) but ≤200 mg/dl (11.1 mmol/l). In the absence of medical record information, we confirmed self-reported T2D cases by performing a 2-h OGTT. The 2-h OGTTs were performed following the criteria of the World Health Organization (WHO) (75 g oral load of glucose). Body mass index (BMI) was calculated as [weight (kg)/height (meter)^2^], and waist-hip ratio (WHR) was calculated as the ratio of abdomen or waist circumference to hip circumference.

The NGT subjects participated in this study were from the same Khatri Sikh community from which the T2D patients were recruited [16]. Majority of the subjects were recruited from the state of Punjab from Northern India. Individuals of South, East and Central Indian origin, or with type -1 diabetes or family member with type -1 diabetes, rare forms of T2D called maturity-onset diabetes of young (MODYs), and secondary diabetes (e.g., hemochromatosis, pancreatitis) were excluded from the study. Of the 386 controls, 180 were normal spouses of diabetic patients, and the remaining 206 were unrelated controls who had no first degree relative affected with T2D, but had other chronic illnesses such as hypertension, coronary heart disease or arthritis. The selection of controls was based on a fasting glycemia < 110 mg/dl or < 6.0 mmol/l or a 2-h glucose <140 mg/dl (< 7.8 mmol/l). Control subjects with HbA1c levels >6.5% were excluded. The average age of NGT controls (mean ± SD) was 50.5 ± 13.8 yrs. Additionally, 22 subjects with IGT were excluded from analyses. In general, Sikhs do not smoke for religious and cultural reasons and about 50% of them were life long vegetarians. Clinical characteristics of the SDS subjects used for this investigation are summarized in Table 1. All blood samples were obtained at the baseline visit and all participants provided a written informed consent for investigations. All SDS protocols and consent documents were reviewed and approved by the University of Oklahoma and the University of Pittsburgh Institutional Review Boards as well as the Human Subject Protection Committees at the participating hospitals and institutes in India. Memorandum of understanding and material transfer agreements for sample sharing and intellectual property rights were signed between collaborating Indian and the US Institutes. The study was approved by the Indian Council of Medical Research, New Delhi and Health Ministry Screening Committee, Union Ministry of Health and Family Welfare, Government of India.

**Table 1:** Clinical characteristics of study population stratified by gender (Mean ± SD)

		**T2D Cases**	**NGT**	
			
	**Gender**	532 (299M/233F)*	**386 (184M/202F)**	*p*value**
Age (yrs)	M*	53. 2 ± 11.1	51.7 ± 15.6	0.006
	F	55.2 ± 11.0	50.8 ± 13.0	0.001
Age at				
diagnosis (yrs)	M	47.5 ± 10.9	--	--
	F	48.2 ± 10.5	--	--
Duration of				
Diabetes (yrs)	M	8.6 ± 7.4	--	--
	F	7.3 ± 6.0	--	--
BMI	M	26.6 ± 4.3	26.6 ± 4.4	0.912
	F	29.0 ± 5.5	27.5 ± 4.9	<0.001
WHR^†^	M	0.99 ± 0.06	0.97 ± 0.07	0.002
	F	0.93 ± 0.07	0.91 ± 0.07	<0.001
Fasting Glucose				
(mmol/l)	M	10.1 ± 3.7	5.4 ± 0.6	<0.0001
	F	10.0 ± 3.5	5.4 ± 0.5	<0.0001
Fasting				
Insulin(IU/ml)	M	9.1 ± 7.9	11.0 ± 9.2	0.057
	F	8.8 ± 7.4	11.5 ± 9.5	0.002
HbA1c (%)	M	8.6 ± 2.4	5.6 ± 0.7	<0.0001
	F	8.6 ± 2.5	5.6 ± 0.7	<0.0001

*M – male, F – female; ^†^Waist to hip ratio; ** Difference between T2D cases and NGT controls

### Metabolic Assays

Quantitation of lipids [total cholesterol, low-density lipoprotein (LDL) cholesterol, high-density lipoprotein (HDL) cholesterol, very low-density lipoprotein (VLDL) cholesterol, triglycerides (TG)], insulin, and creatinine levels were estimated in serum. HbA1c was quantified in whole blood. Lipids were quantified by using standard enzymatic methods (Roche, Basel, Switzerland). HbA1c levels were determined by turbidometric inhibition immunoassay (Tina Quant; Roche, Basel, Switzerland). Creatinine was quantified by using kinetic colorimetric assay (Roche, Basel, Switzerland). Insulin was measured by radio-immuno assay (Diagnostic Products, Cypress, USA). All quantitative parameters were determined by following manufacturer's instructions using Hitachi 902 auto-analyzer (Roche, Basel, Switzerland).

### SNP Genotyping

DNA was extracted from buffy coats using QiaAmp blood kits (Qiagen, Chatworth, CA) or by the salting out procedure [18]. Genotyping for all investigated SNPs was performed using TaqMan Pre-Designed or TaqMan Made-to Order SNP genotyping assays from Applied Biosystems (ABI, Foster City, USA). TaqMan genotyping reactions were performed on ABI 7900 genetic analyzer using 2 ul of (10 ng/ul) of genomic DNA following manufacturer's instructions. Florescence was detected on an ABI prism 7900HT sequence detection system (ABI, Foster City, USA). Genotypes were scored by analyzing data on both real-time as well as allele discrimination assay platforms using SDS software provided by the ABI. Data quality for SNP genotyping was checked by establishing reproducibility of control DNA samples. For quality control, 30 replicate positive controls and 8 negative controls were included in each run to match the concordance, and the discrepancy in the concordance was < 0.2%. Genotyping success rate ranged from 96%–99.8% for all the investigated SNPs except for *CDKN2A *(rs 10811661) for which the success rate was 88.5% due to technical difficulties.

### Statistical Analysis

We evaluated Hardy-Weinberg equilibrium using a one-degree of freedom goodness-of-fit test separately among cases and controls using Pearson chi-square test. The allele frequencies in T2D cases were compared to those in controls using chi-square test or Fisher's exact probability test, where appropriate. Statistical evaluations for testing genetic effects of association between the case-control status and each individual SNP, measured by the odds ratio (OR) and its corresponding 95% confidence limits were estimated using unconditional logistic regression before and after adjusting for age, gender, and other covariates. Association analyses were performed assuming a dominant, recessive and co-dominant effect for each polymorphism using SNPassoc [19]. In all analyses, the common homozygote genotype in the control population was defined as the reference category. The likelihood ratio test was used to test the effect of each SNP at the nominal 5% significance level. Akaïke's information criterion [20] was also used to select the best genetic model for each SNP. We also performed multiple linear regression analysis to examine the impact of these variants on quantitative risk variables of T2D, including fasting insulin, glucose, and lipid levels. Skewed variables for the continuous traits (e.g., triglycerides, cholesterol, LDL, glucose) were log-transformed before statistical comparisons. Significant covariates for each dependent trait were identified by using Spearman's correlation and step-wise multiple linear regression with an overall 5% level of significance.

## Results

We genotyped nine SNPs, including the six SNPs from newly emerged gene regions recently identified by multiple GWA studies [5-7,9,21], in a case-control cohort of Asian Indian Sikhs consisting 918 subjects including, 532 T2D cases and 386 NGT controls matched for ethnicity and geographic origin. Table 1 shows the clinical and physical characteristics of the study subjects separated by gender. The information on chromosomal position and genomic locations of the investigated SNP, base change, genotype, allele frequencies, and ORs is summarized in Tables 2. Genotype distribution for all investigated SNPs was in Hardy-Weinberg equilibrium in both cases and controls. The genes containing SNPs that showed strong association with T2D in our sample were *PPARG2 *(rs18081282), *IGF2BP2 *(rs 4402960), *TCF7L2 *(rs10885409), and *FTO *(rs9939609). The frequencies of major alleles (at risk allele) of *PPARG2 *and minor alleles (at risk allele) of *IGF2BP2*, *TCF7L2 *and *FTO *were significantly higher among diabetics than controls (Tables 2). The adjusted ORs under a recessive model provided a strongest evidence of association for *PPARG2 *[OR 0.12; 95% CI (0.03–0.52); p = 0.005] showing protective association with T2D for the less common Ala (G) allele. On the other hand, dominant models suggested strongest association for the other three genes: OR for *IGF2BP2 *[1.37; 95% CI (1.04–1.82); p = 0.027], OR for *TCF7L2 *[1.64; 95% CI (1.20–2.24); p = 0.001] and OR for *FTO *[1.46; 95% CI (1.11–1.93); p = 0.007] when T2D subjects were compared with NGT. However, our study could not replicate the significant association of the remaining five SNPs reported in earlier studies by analyzing each SNP individually.

**Table 2:** Candidate gene polymorphisms and type 2 diabetes risk in Khatri Sikhs; comparing NGT vs. T2D

						ODDS RATIO (ORs*)		
**GENE/SNP**	**CHROMO SOME**	**POSITION^ψ^**	**GENOTYPE**	**NGT (%)**	**T2D (%)**	**CO-DOMINANT **	**DOMINANT **	**RECESSIVE **
						OR (95% CI)	OR (95% CI)	OR (95% CI)
***PPARG2***	3	12336825						
rs 1801282			CC	286 (76.5)	414 (80.4)	CC vs. CG, GG	CC vs. CG+GG	CC+CG vs. GG
			CG	78 (20.8)	99 (19.2)	0.96 (0.68–1.35)	0.84 (0.61–1.17)	0.12 (0.03–0.52)
			GG	10 (2.7)	2 (0.4)	0.11 (0.03–0.51)		
			Allele C	0.87	0.90	CG; p = 0.824	p = 0.300	p = 0.005
			Allele G	0.13	0.10	GG; p = 0.005		
***IGF2BP2***	3	186994381						
rs 4402960			GG	150 (38.9)	160 (31.1)	GG vs. GT, TT	GG vs. GT+TT	GG+GT vs. TT
			GT	173 (44.8)	262 (51.0)	1.36 (1.01–1.84)	1.37 (1.04–1.82)	1.18 (0.82–1.68)
			TT	63 (16.3)	92 (17.9)	1.42 (0.95–2.10)		
			Allele G	0.61	0.57	GT; p = 0.041	p = 0.027	p = 0.372
			Allele T	0.39	0.43	TT; p = 0.087		
***CDK5***	6	20769229						
rs 7754840			GG	203 (55.2)	281 (53.7)	GG vs. CG, CC	GG vs. CG+CC	GG+CG vs. CC
			CG	134 (36.4)	196 (37.5)	1.04 (0.78–1.40)	1.06 (0.80–1.37)	1.10 (0.68–1.78)
			CC	31(8.4)	46 (8.8)	1.12 (0.68–1.83)		
			Allele G	0.73	0.55	CG; p = 0.771	p = 0.688	p = 0.692
			Allele C	0.27	0.45	CC; p = 0.655		
***SLC30A8***	8	118253964						
rs 13266634			CC	188 (53.9)	290 (54.5)	CC vs. CT, TT	CC vs. CT+TT	CC+CT vs. TT
			CT	135 (38.7)	195 (36.6)	0.95 (0.71–1.27)	0.99 (0.75–1.30)	1.23 (0.74–2.02)
			TT	26 (7.4)	47 (8.8)	1.21 (0.72–2.04)		
			Allele C	0.73	0.73	CT; p = 0.718	p = 0.946	p = 0.424
			Allele T	0.27	0.27	TT; p = 0.468		
***CDKN2A***	9	22124094						
rs 10811661			TT	232 (78.6)	427 (82.7)	TT vs. CT, CC	TT vs. CT+CC	TT+CT vs. CC
			CT	61 (20.6)	87 (16.9)	0.85 (0.58–1.23)	0.84 (0.58–1.21)	0.54 (0.08–3.93)
			CC	2 (0.7)	2 (0.4)	0.51 (0.07–3.73)		
			Allele T	0.89	0.91	CT; p = 0.386	p = 0.343	p = 0.545
			Allele C	0.11	0.09	CC p = 0.511		
***HHEX***	10	94452862						
rs 1111875			TT	120 (32.7)	154 (30.0)	TT vs. CT, CC	TT vs. CT+CC	TT+CT vs. CC
			CT	166 (45.2)	242 (47.0)	1.28 (1.02–1.90)	1.22 (0.93–1.60)	1.10 (0.82–1.47)
			CC	81 (22.1)	118 (23.0)	1.16 (0.80–1.67)		
			Allele T	0.55	0.54	CT; p = 0.107	p = 0.147	p = 0.529
			Allele C	0.45	0.46	CC; p = 0.438		
***TCF7L2***	10	114798062						
rs 10885409			TT	118 (31.7)	112 (21.8)	TT vs. TC, CC	TT vs. TC+CC	TT+TC vs. CC
			CT	169 (45.4)	257 (50.0)	1.59 (1.14–2.23)	1.64 (1.20–2.24)	0.76 (0.55–1.03)
			CC	85 (22.8)	145 (28.2)	1.72 (1.18–2.53)		
			Allele T	0.54	0.47	TC; p = 0.006	p = 0.001	p = 0.078
			Allele C	0.46	0.53	CC; p = 0.003		
***KCNJ11***	11	17366148						
rs 5219			CC	148 (39.6)	226 (42.5)	CC vs. CT. TT	CC vs. CT+TT	CC+CT vs. TT
			CT	169 (45.2)	247 (46.4)	0.97 (0.73–1.30)	0.90 (0.69–1.19)	0.71 (0.48–1.06)
			TT	57 (15.2)	59 (11.1)	0.70 (0.46–1.08)		
			Allele C	0.66	0.62	CT; p = 0.835	p = 0.458	p = 0.091
			Allele T	0.34	0.38	TT; p = 0.110		
***FTO***	16	52378028						
rs 9939609			TT	175 (49.6)	205 (39.9)	TT vs. AT, AA	TT vs. AT+AA	TT+AT vs. AA
			AT	137 (38.8)	244 (47.6)	1.51 (1.30–3.03)	1.46 (1.11–1.93)	1.08 (0.71 – 1.64)
			AA	41 (11.6)	64 (12.5)	1.32 (0.84–1.93)		
			Allele T	0.69	0.64	AT; p = 0.006	p = 0.007	p = 0.737
			Allele A	0.31	0.36	AA; p = 0.227		

*ORs were adjusted for age, sex, and BMI; ^ψ ^Position of SNPs on chromosome has been taken from NCBI (Genome Build 36.3)

To explore the possible mechanisms of action of these variants for affecting T2D susceptibility, we tested the association of these SNPs with the T2D-related quantitative phenotypes (height, weight, BMI, WHR, insulin, creatinine, total cholesterol, LDL-cholesterol, VLDL-cholesterol, HDL-cholesterol, and triglycerides) using multiple linear regression analysis in NGT after adjusting for the effects of age, sex, BMI, disease status, and medication. As shown in Table 3, the 'C' (risk) allele of *CDK5 *variant was significantly associated with decreased HDL-cholesterol levels in NGT (p = 0.005) as well as combined (NGT+T2D) sample (p = 0.005). The less common 'C' (risk) allele of *TCF7L2 *polymorphism also showed significant association with increased levels of LDL-cholesterol in NGT (p = 0. 010) and total and LDL-cholesterol in combined sample (p = 0.008; p = 0.003, respectively).

**Table 3:** SNPs with significant association to quantitative phenotypes of T2D

**Gene/SNP**	**Risk Allele**	**Trait associated with T2D**	**R-Square**	**p (adjusted)**
**NGT Subjects**^¥^				
***CDK5***	C	HDL-cholesterol ↓ Φ	0.021	0.005
rs 7754840				
***TCF7L2***	C	Total-cholesterol ↑	0.004	0.083
rs 10885409		LDL-cholesterol↑	0.016	0.010

Combined**				
**NGT, T2D**				
***CDK5***	C	HDL-cholesterol ↓	0.021	0.005
rs 7754840				
***TCF7L2***	C	Total-cholesterol↑	0.009	0.008
rs 10885409		LDL-cholesterol↑	0.012	0.003

^¥ ^Multiple linear regression analyses were adjusted for age, sex, BMI, and medication; **Multiple linear regression analyses were adjusted for age, sex, BMI, disease status, and medication; Φ Arrows indicate risk-allele associated increase or decrease in quantitative trait.

## Discussion

In this investigation, we have carried out an unbiased replication of SNPs associated with T2D susceptibility in recently published multiple GWA studies [5-8] in our independent dataset of Asian Indian origin. Two of these SNPs belong to highly replicated biological candidate genes *PPARG2 *(Pro12Ala; rs 1801282) and *KCNJ11 *(Glu23Lys; rs 5219) that produced unequivocal evidence of their involvement in T2D in several independent studies performed in different data sets [22,23]. Moreover, these two genes are also targets for anti-diabetic therapies. *PPARG2 *encodes a transcription factor involved in adipocyte differentiation and is the target of thizolodinedione class of drugs used for T2D [24] and *KCNJ11 *is the target of sulphonylurea class of drugs extensively used for treating neonatal diabetes [25,26]. The remaining seven variants (*IGF2BP2*, *CDK5*, *SLC30A8*, *CDKN2A*, *HHEX, TCF7L2 *and *FTO*) evaluated in this study are from the recently identified gene regions that provide convincing evidence of their involvement in T2D in fine mapping and multiple GWA studies.

We have identified a significant association of *PPARG2 *(rs1801282; p = 0.005), *IGF2BP2 *(rs 4402960; p = 0.027) and *FTO *(rs 9939609; p = 0.007) variants with T2D along with the *TCF7L2 *variant (rs10885409; p = 0.001) in our Asian Indian population. The protective association of the less common 'Ala' allele at codon 12 of *PPARG2 *gene has been consistently reproduced in multiple independent studies conducted in different populations [22,27-31] with few exceptions of small studies e.g. Oji-Cree [32] and Czech [33], where the Ala allele was shown to be associated with T2D risk. The protective association of Ala allele was also not confirmed in a South Indian population [34]. The observed difference of association within Asian Indian populations is important in view of the extensive diversity present between Indian populations [35-37]. *IGF2BP2 *belongs to a family of three mRNA binding proteins; the human growth hormone gene and insulin like growth factor 1 and 2 genes. It is involved in pancreas development, growth and stimulation of insulin action [38,39]. We have replicated a significant association of *IGF2BP2 *(rs 4402960) SNP reported earlier in three different GWA studies [5,11,12], meta analysis of three GWA studies [7], and also in Japanese population [40]. However, no association of this variant was noticed with any quantitative trait related to glucose homeostasis or lipid metabolism.

The *FTO *gene is of unknown function that was originally cloned as a result of identification of fused toe mutant mouse [41]. Recently, the recombinant murine fto has been shown to be involved in nucleic acid methylation by catalyzing Fe (II) – and 2-oxoglutarate (2OG)-dependent dioxygenases [42,43]. This locus is associated with childhood and adult obesity in several European and US populations [9,44,45]. The *FTO *variant (TA A) (rs 9939609) has also been shown to be strongly associated with T2D risk (OR 1.27; p = 5 × 10^-8^) in GWA scan performed in UK population [9]. Our study also confirmed significant association of *FTO *(rs 9939609) variant with T2D in this Asian Indian sample. But the mechanism by which it relates to obesity and T2D in humans is still unclear. Studies have shown that T2D risk associated with 'A' allele of this variant (rs 9939609) is strongly mediated by BMI, and has been shown to potentially affect mass rather than height [9]. In contrast, in this Asian Indian sample, the T2D risk does not seem to be mediated by BMI or weight, as by excluding BMI, the risk associated with this variant did not disappear, as reported in other studies [9,40]. Perhaps the ethnic difference could be responsible for this difference. Moreover, BMI is not considered an accurate measure for obesity, especially in the populations from South Asia where at a given BMI, their muscle mass is low and visceral and subcutaneous fat is increased [46-48]. Therefore, measures of BMI or waist and hip may not reveal accurate estimates of total fat.

The importance of *TCF7L2 *as diabetes gene is now very well established across ethnicities but many more genes for diabetes especially in non-white ethnicities still remain to be identified. It is interesting to observe that the risk alleles found to be associated with T2D in this Asian Indian population in four significant SNPs (*PPARG2, IGF2BP2, TCF7L2 and FTO*) were consistent with those reported in GWA studies in Caucasians and Japanese [5,7,11,40]. However, it is still unclear how these loci contribute to T2D risk as none of these variant was found to influence insulin or fasting glucose levels in this study. More functional studies would be needed to clearly define the role of these variants in glucose homeostasis. Perhaps the causal variants in these genes controlling insulin secretion and insulin resistance are yet to be identified. With regard to remaining loci, our study did not confirm the previous reported association of variants (*KCNJ11*, *CDK5*, *SLC30A8*, *CDKN2A *and *HHEX*) when each SNP was analyzed separately. With the exception of *CDK5 *and *TCF7L2*, no other gene variant showed significant association with any diabetes-related quantitative traits. The 'C' (risk) allele of *CDK5 *variant was associated with significantly reduced HDL levels (p = 0.005) in this sample. The possibility of false positive results in our sample cannot be ruled out because of relatively small size of our cohort. Also, while our manuscript was being reviewed, publication of two large meta-analysis studies on lipid traits in subjects with coronary artery disease and T2D did not report this locus affecting lipid traits in Caucasians [49,50]. However, as we previously reported [15], five SNPs in the *TCF7L2 *gene (rs 7901695, rs7903146, rs11196205, rs10885409, and rs 12255372) revealed significantly elevated gene-dosage effects on total cholesterol, LDL cholesterol and VLDL cholesterol levels in this Khatri Sikh sample. It is therefore possible that the observed association with quantitative lipid levels could be population-specific and may not be present in other ethnicities. Replication of these findings in a larger sample of Asian Indian origin would be required confirm these findings.

On the other hand, the absence of positive association with T2D in remaining loci may suggest that the genetic variation in some loci could be restricted to a particular genetic background and environment and the contribution of these loci in Indian population may be minor. Alternatively, different SNPs within the same gene could be contributing to the risk and could be population specific. Therefore, further SNP screening and deep sequencing of these genes would be required to identify putative ethnicity-specific functional variants in these novel candidates.

## Conclusion

Our study in Asian Indian Sikhs has successfully replicated the association of four of nine loci recently reported to be associated with T2D in Caucasians. To our knowledge, this is the first report describing the role of some recently emerged loci with T2D in a population of Asian Indian origin from North. Further investigations are warranted to cross-validate these findings in other larger datasets. Functional studies would help understand the pathway-based implications of these important loci in T2D pathophysiology in different ethnicities.

## Competing interests

The authors declare that they have no competing interests.

## Authors' contributions

All authors have read and approved the final manuscript. LO carried out the molecular genetic studies, acquisition of data, SH participated in statistical analysis, JS participated in recruitment of study subjects, study design and manuscript editing, SKR participated in recruitment of study subjects, study design and manuscript editing, GSW participated in recruitment of study subjects, study design and manuscript editing, NKM participated in study design, sample collection and manuscript drafting, JJM helped in quality control, and manuscript drafting, REF and MIK helped in conceptualizing the project, data interpretation, intellectual input and manuscript drafting, SKN carried out statistical analysis, data interpretation, DKS is principal investigator and coordinator of the project, involved in conceptualizing the project, study design, genotyping data quality control, analysis, interpretation, manuscript drafting and finalizing the manuscript.

## Pre-publication history

The pre-publication history for this paper can be accessed here:


